# Sternal cavernous hemangioma and reconstruction of the anterior chest wall: a case report

**DOI:** 10.1186/s12893-020-00961-y

**Published:** 2020-11-19

**Authors:** Liliana Fernández-Trujillo, Saveria Sangiovanni, Eliana I. Morales, Valeria Marin, Luz F. Sua, Mauricio Velasquez

**Affiliations:** 1 Department of Internal Medicine, Pulmonology Service, Interventional Pulmonology, Fundación Valle del Lili, Avenida Simón Bolívar, Carrera 98 # 18-49, Tower 6, 4th Floor, Cali, 7600032 Colombia; 2 Faculty of Health Sciences, Universidad Icesi, Calle 18 # 122-135, Cali, 7600032 Colombia; 3 Clinical Research Center, Fundación Valle del Lili, Carrera 98 # 18-49, Cali, 7600032 Colombia; 4 Department of Internal Medicine, Pulmonology Service, Fundación Valle del Lili, Carrera 98 # 18-49, Cali, 7600032 Colombia; 5 Faculty and Postgraduate School of Dentistry, Universidad del Valle, Calle 4B # 36-00, Cali, Colombia; 6 Department of Innovation and Technology, HUMANBX S.A.S, Carrera 65 # 1A-93, Cali, Colombia; 7 Department of Pathology and Laboratory Medicine, Fundación Valle del Lili, Carrera 98 # 18-49, Cali, 7600032 Colombia; 8 Department of Surgery, Thoracic Surgery Service, Fundación Valle del Lili, Carrera 98 # 18-49, Cali, 7600032 Colombia

**Keywords:** Sternal cavernous hemangioma, Primary sternal tumors, Surgical resection and reconstruction, Case report

## Abstract

**Background:**

The sternum is considered an unusual tumor site, corresponding to 15% of all thoracic wall tumors. Primary sternal tumors are even rarer and most commonly malignant. We present the case of a young man who consulted with a painful sternal mass, which after its resection is confirmed to be a cavernous hemangioma.

**Case presentation:**

A 39-year-old man, with unremarkable medical history besides a 2-year-long sternal pain, non-irradiated, which worsens over the last few months and is accompanied by the appearance of a sternal palpable mass. On physical exam, there was a bulging of the sternal manubrium, with no inflammatory changes. Thoracic CT scan shows an expansive and lytic lesion of the sternum, compromising the manubrium and extending to the third sternocostal joint, without intrathoracic compromise nor cleavage plane with mediastinal vascular structures. The patient is taken to resection of the mass and sternal reconstruction using prosthetic material and pectoral and fasciocutaneous muscular flaps. Histopathological findings: cavernous hemangioma with negative borders and no other malignant findings.

**Conclusions:**

Sternal hemangiomas can cause defects in the bone structure and show an expansive growth, challenging the differentiation between a benign or malignant lesion. Therefore, they should be considered malignant until shown otherwise. Management involves radical surgery with curative purposes and posterior reconstruction to improve quality of life, as shown with our patient.

## Introduction

The sternum is considered an unusual tumor site, with an overall incidence of 15% of all tumors of the chest wall [1]. Primary sternal tumors are even rarer and most commonly malignant [2]. Within benign lesions, hemangiomas are described, but they typically affect soft tissues and when bone tissue is compromised, it tends to be the skull or vertebrae [1, 3]. Management is mainly surgical, with wide excisions and reconstruction being the standard of care. Furthermore, the prognosis of patients with benign tumors of the chest wall is excellent after excision [1]. We present the case of a young man who presents to a high complexity institution, with a painful sternal mass, that is diagnosed as a cavernous hemangioma after complete resection.

## Case presentation

A 39-year-old man, without a significant personal history, who consults to the thoracic surgeon for a 2-year history of non-irradiated sternal pain, exacerbated by intense exercise. In recent months, his quality of life worsened and a palpable sternal mass became evident. On physical examination he had, blood pressure 120/80 mm Hg in both arms, heart rate 82 beats per minute, respiratory rate 12 breaths per minute, SO_2_ 97%, temperature 36.5 °C, there was no jugular engorgement or neck masses, upon inspection of the thorax, in the sternal area, there was a mass of more or less 10 cm, without inflammatory changes or ulcers, with a hard texture on palpation, without pulsation or collateral circulation on it, the heart was rhythmic without murmurs or gallops, respiratory sounds were normal, he had no abdominal masses, lower limb edema, or neurological deficit. He assists with a bone scan that showed osteoblastic changes in the sternal manubrium, suggestive of tumor vs infectious process. Additionally, he had a thoracic computerized tomography (CT) scan that evidenced an expansive lytic lesion, mainly in the manubrium but extending into to the third costoesternal joint, without compromising intrathoracic organs and with cleavage plane of the mediastinal vascular structures. There were no other associated lesions.

**Fig. 1:**
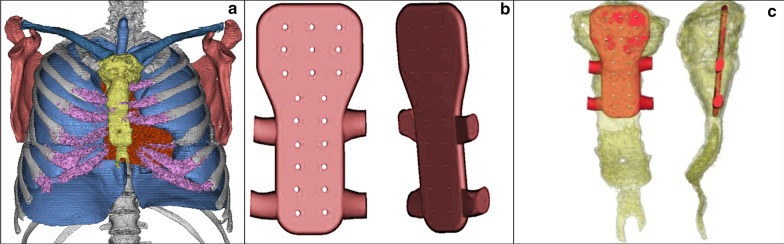
**a** 3D thoracic model based on the tomographic images of the patient, using the Mimics software (Version 21.0, Materialise, Inc., Leuven, Belgium), from which the tumor and rib resection was planned, according to the standard of leaving a clean margin of at least 30 mm. **b**, **c** Model width: 4 mm, widest lateral border: 56 mm, highest lateral border: 105 mm. There was no need for ramifications to the sternoclavicular joints because of the chosen surgical technique, therefore the model had only 4 anchor points for rib attachment

**Fig. 2.**
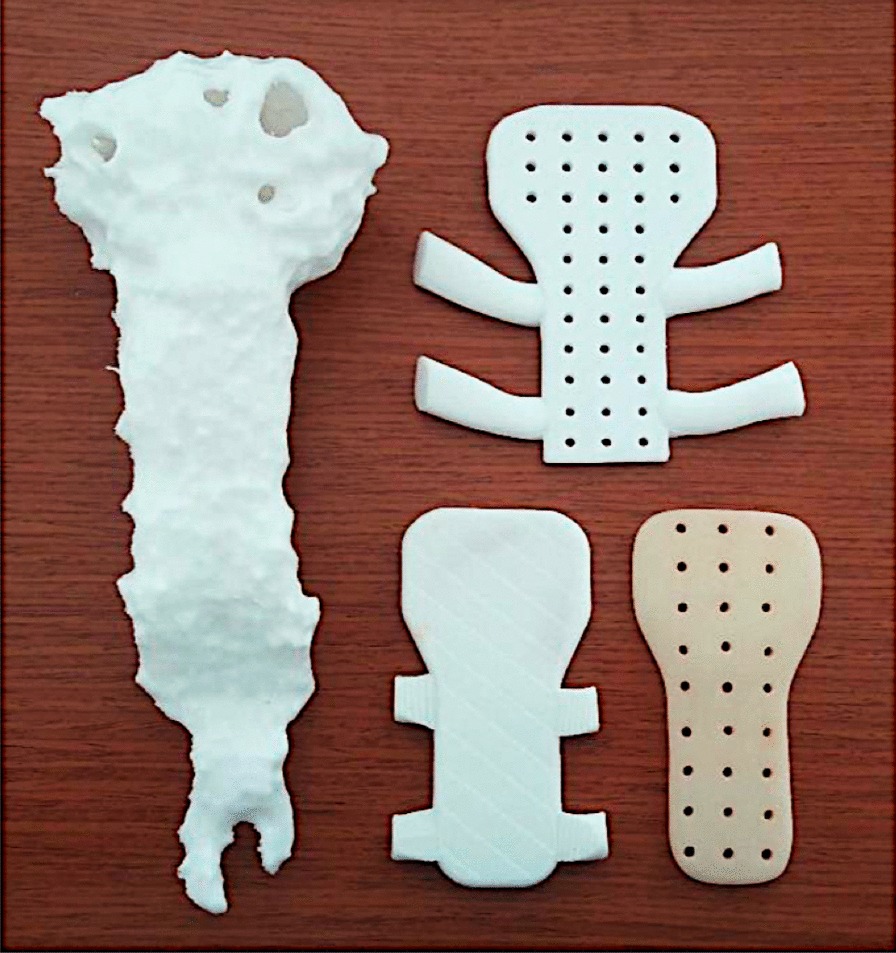
3D printing of the sternum and several bio models of prosthetic prototypes from which one was chosen

In a multidisciplinary board, the decision was made to perform a sternum, rib, and clavicle resection, followed by reconstruction. A literature search was carried out to find the best way to reconstruct the sternum, finding several alternatives, so we chose to study in-depth those that we had available in our country. We had to choose between using Polyether ether ketone (PEEK), poly-methyl-methacrylate (PMMA), or titanium. We had experience with PEEK and PMMA in the past, being the latter easier to manipulate intraoperatively, so we decided to go with it. After making the decision we looked into the image archive of our institution for thoracic CT images of patients with similar physical characteristics to our case, to plan the size and details of the prosthesis to be built. Then we compared the designed sternum with the patient's sternum to see if it would fit properly. For this process, the Mimics Innovative Suite software (Mimics Medical 21.0 and 3—Matic Medical 13.0) and then a 3D printer was used to obtain the bio model, from which the prosthesis was made (Figs. 1, 2).

**Fig. 3:**
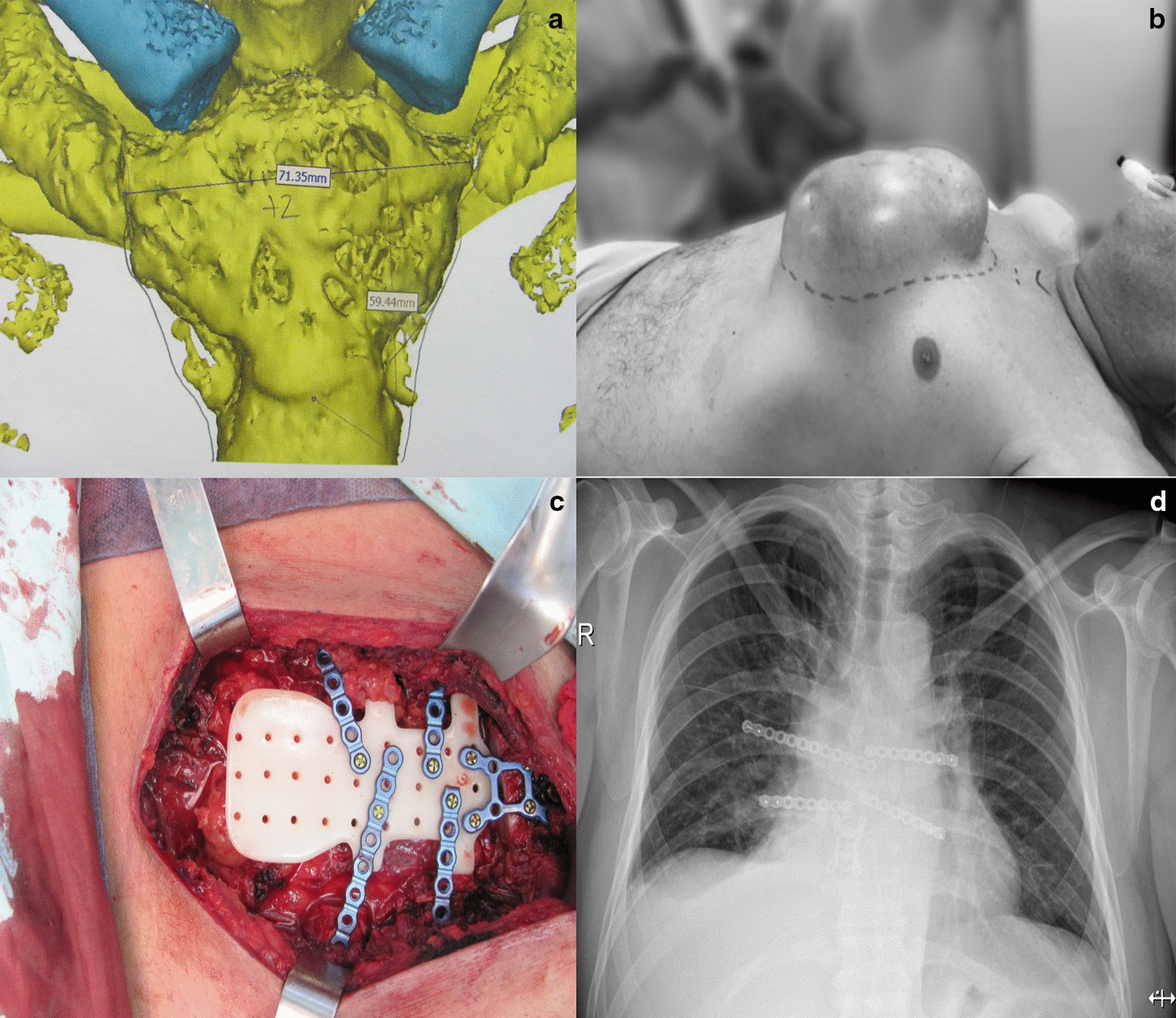
**a** 3D model to plan the sternal reconstruction. **b** Image of the mass protruding to the anterior wall of the thorax, over the sternal area, previous to surgery. **c** Surgical procedure with the installed prosthesis on the sternal space. **d** Post-surgical thoracic x-ray where the fixation elements of the prosthesis are evidenced in a good position

**Fig. 4:**
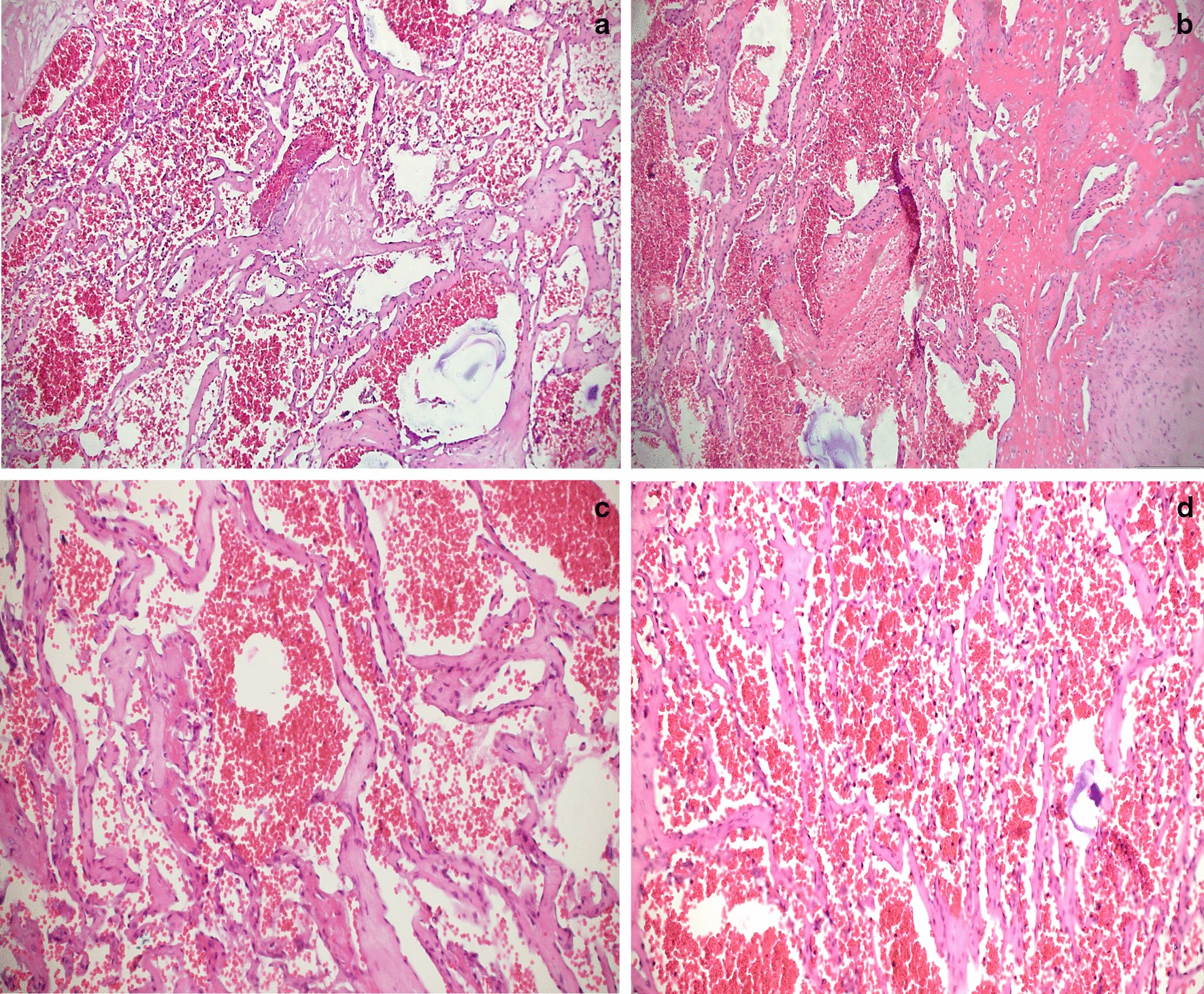
**a**, **b** H&E-10×. **c**, **d** H&E-20×. Vascular lesion composed of tortuous and dilated vascular channels, lined by endothelial cells without cytologic atypia or mitotic figures. Abundant erythrocytes in the interior of the vessels

Intraoperative findings were that of a large tumor of the sternal manubrium without the involvement of the clavicles or ribs bilaterally. A sternal resection was performed from the third intercostal space until the union between the first intercostal space and the clavicles. A thoracic wall reconstruction using the PMMA prosthesis and the osteosynthesis material to fix it to the thorax was performed, trying foremost to protect the physiological function of the sternum in the respiratory system. A plastic surgeon covered the material using pectoral and fasciocutaneous muscle flaps (Fig. 3). The patient was then transferred to hospitalization, where he remained for a total of 7 days. He did not require admission to the intensive care unit or developed any infectious complications. At day 5 he initiated physical therapy. Histopathological results showed a cavernous hemangioma with negative borders and no other signs of malignancy (Fig. 4). Follow up appointments with the thoracic surgeon and plastic surgeon were programmed for 48 h, 1–3–6 months after hospital discharge, and then in a biannual basis, with a chest radiography (x-ray). After 4 months, the patient had full mobilization of upper extremities with no compromise of sternoclavicular joint and normal respiratory function. At 10 months he was considered to be ready to resume work activities. His last appointment was on December 2019, showing excellent evolution, painless, no physical disabilities, and no evidence of mass regrowth.

## Discussion and conclusions

Primary chest wall tumors are very uncommon, corresponding to less than 2% of all new primary tumors. Furthermore, only 15% of the bony chest wall tumors originate from the sternum, and the exceeding 85% arise from the ribs. Osseous tumors tend to show mixed cellularity, in addition to expansive growth, which makes it difficult to establish its benign nature. Any sternal mass in an adult should be considered malignant until proven otherwise [4]. Specifically, hemangiomas arise from vascular tissue and are extremely rare in the sternum; there are only a couple of case reports in the literature. They are composed of thin-walled and dilated vessels that can arise from the chest wall, as in this case, or from intrathoracic structures. Additionally, they can be classified according to the predominant type of vascular structure into capillary, cavernous, arteriovenous, or venous [5].

Typical clinical presentation includes nonspecific pain, that sometimes becomes evident after chest trauma, and on some occasions a palpable mass [1, 4]. On imaging, osseous tumors are easily seen on chest-rays (> 20%), yet the differentiation between malignant and benign chest tumors is not always feasible using radiographic criteria alone. Cross-sectional imaging techniques such as thoracic CT or magnetic resonance imaging (MRI) are more precise at characterizing the tumors; multidetector CT is great at revealing the size and extent of the mass, as well as the tissue of origin, morphology, composition, and vascularity when contrast is administered. MRI is the ideal modality due to its ability to contrast soft tissue, offers better spatial resolution and differentiation from infectious and/or inflammatory processes [4, 5]. In our case, the patient came to us with a CT scan, which was used to characterize and plan the tumor resection. No further images were made, considering the economic resources required to perform an additional image versus the additional information it would provide.

As for the surgical procedure, wide excision is the standard of care, especially in the case of malignant tumors to achieve local control. Nevertheless, the approach is similar for all primary sternal tumors. Sternal resection must be wide enough to obtain a clear margin of at least 3 cm to minimize the risk of recurrence. A partial sternectomy should be done when the mass is limited to the manubrium as in our case. Depending on the extent of the lesion and the compromise of the superior portion of the manubrium and clavicles, then a total or subtotal sternectomy should be chosen. Involvement of the anterior chest wall and chest cavity structures must be carefully checked upon and managed accordingly; lung and mediastinal structures have to be excised in bloc, while vascular structures are ligated and excised. Following the sternal resection, chest tubes must be placed in each pleural cavity [6]. It is of foremost importance to highlight the impact a chest wall resection poses on pulmonary function when not done properly; it is obvious that the greater the resection, the greater the impact on lung function. However, studies have shown that large resections with rigid stabilizations using prosthetic reconstruction don't impact negatively on postoperative mechanical ventilation time, pulmonary function, or patient discomfort [7].

A well-done reconstruction of the skeletal defect is imperative to maintain adequate chest wall stability. There is an extensive list of prosthetic materials for chest wall reconstruction in the market; ideally, they should be rigid enough to avoid paradoxical chest wall motion but malleable enough to allow chest movement, physically and chemically inert, should allow growth of tissue, must be radiolucent to decrease image artifacts, sterile, resistant to infection and inexpensive. No prosthetic material meets all these criteria and a combination of them is required to get the best results [7]. They can be divided into synthetic materials, biological meshes, and osteosynthesis systems. Synthetic meshes and patches can be flexible such as polyglactin mesh, polypropylene mesh (for example, Marlex mesh), or nonabsorbable synthetic polytetrafluorethylene (PTFE) patch or rigid like PMMA. That being said, flexible meshes are easy to manipulate and achieve a tight closure, they are well incorporated into the tissues. However, its permeability, except in the case of PTFE patches, make it difficult to control pleural effusions. Also, in the case of getting contaminated meshes are difficult to remove. For bigger defects, as in our case, a more rigid material is required. PMMA is most commonly used, sandwiched between two layers of mesh, as explained earlier. Although this technique provides great chest wall stability and the best coverage of mediastinal structures, it has been associated with higher rates of complications like seromas, hematomas, and infections, requiring removal of the material [6–10]. Regarding, reconstruction with bioprosthetic materials (cadaveric human dermis, porcine dermis, bovine pericardium, etc.), they have the advantage of being revascularized over time and remodeled into autologous tissue, plus they might be more resistant to infections. However, the evidence is scant regarding its use for chest wall reconstruction [7]. Finally, osteosynthesis systems, which need to be used in combination with the other materials, can allow a more physiologic rib movement when used for bridging the defects. On the downside, they can break or get displaced, and as they have less tissue ingrowth when compared to meshes, they can become infected over time, needing to be removed [6, 7, 9].

Although we did choose PMMA, our approach differs widely from the PMMA sandwich technique. Currently, with the possibility of 3D printing, materials such as a PMMA or PEEK are taking the lead in bone reconstruction since they can be designed and printed to suit the patient. PEEK implants are also a great option due to good biocompatibility, biomechanical properties due to similar tensile strength to sternum and ribs, stability, and radiolucency [11]. Our team chose PMMA over PEEK since it is easier to maneuver intraoperatively.

Despite the concern of choosing an infection resistant prosthetic material, the main source of complications is not the prosthesis itself, or the wound, but pulmonary infections, mainly pneumonia. Pulmonary complications, which are the main cause of mortality, may warrant not only antibiotics but prolonged mechanical ventilation and tracheostomy [6]. Fortunately, our patient did not suffer from local or respiratory complications. Follow up is recommended with thoracic CT scans at 3 and 6 months and then 1 year later for malignant tumors but in the case of benign lesions, follow up can be done with chest x-rays alone [6].

In conclusion, bony chest wall tumors are extremely rare, even more when they compromise the sternum, as is the case of sternal cavernous hemangiomas. Due to the tumor's expansive growth and often mixed cellularity, they should always be considered malignant. Wide excision is the surgical gold standard, along with an excisional biopsy to achieve a histopathological diagnosis. Skeletal reconstruction is fundamental for maintaining the chest wall's stability and improving the patient's quality of life. A multidisciplinary approach with pulmonologists, thoracic surgeons, plastic surgeons, and biomedical engineers is needed to ensure the best possible outcome.

## Data Availability

All data and materials are available for sharing if needed.

## Abbreviations

CT scan: Computed tomography scan
x-ray: Radiography
3D: Three-dimensional
PMMA: Polymethyl methacrylate
MRI: Magnetic Resonance Imaging
PTFE: Polytetrafluoroethylene
PEEK: Polyether ether ketone
